# Src Kinase Conformational Activation: Thermodynamics, Pathways, and Mechanisms

**DOI:** 10.1371/journal.pcbi.1000047

**Published:** 2008-03-28

**Authors:** Sichun Yang, Benoît Roux

**Affiliations:** Department of Biochemistry and Molecular Biology, Gordon Center for Integrative Science, The University of Chicago, Chicago, Illinois, United States of America; University of California San Diego, United States of America

## Abstract

Tyrosine kinases of the Src-family are large allosteric enzymes that play a key role in cellular signaling. Conversion of the kinase from an inactive to an active state is accompanied by substantial structural changes. Here, we construct a coarse-grained model of the catalytic domain incorporating experimental structures for the two stable states, and simulate the dynamics of conformational transitions in kinase activation. We explore the transition energy landscapes by constructing a structural network among clusters of conformations from the simulations. From the structural network, two major ensembles of pathways for the activation are identified. In the first transition pathway, we find a coordinated switching mechanism of interactions among the αC helix, the activation-loop, and the β strands in the N-lobe of the catalytic domain. In a second pathway, the conformational change is coupled to a partial unfolding of the N-lobe region of the catalytic domain. We also characterize the switching mechanism for the αC helix and the activation-loop in detail. Finally, we test the performance of a Markov model and its ability to account for the structural kinetics in the context of Src conformational changes. Taken together, these results provide a broad framework for understanding the main features of the conformational transition taking place upon Src activation.

## Author Summary

Src tyrosine kinases are large protein molecules that play an important role in the regulation of cellular growth and proliferation. In doing so, Src kinases have the ability to affect the activity of other proteins inside the cell by turning them “on” or “off.” Dysfunctional Src kinase activity has been associated with many human diseases, most importantly cancer, which makes them important targets for therapeutic intervention. To understand how a Src kinase molecule is able to change its shape (conformation) and switch between its active or inactive states, we constructed a computer model. The results from the model provide a broad conceptual framework for interpreting the main features of the change of protein conformation taking place upon Src activation. It is our hope that these results will help design new experiments to refine our understanding of the activation of Src kinases.

## Introduction

The nonreceptor tyrosine kinases of the Src-family are large allosteric enzymes involved in signaling pathways, regulating cell growth and proliferation [1]–[4]. These enzymes have the ability to undergo large conformational changes, thereby “switching” between different inactive and active “states” in response to either intracellular or extracellular signals. The key role that these kinases play in the onset of many human diseases, particularly cancer, makes them important targets for therapeutic intervention [5].

**Figure 1:**
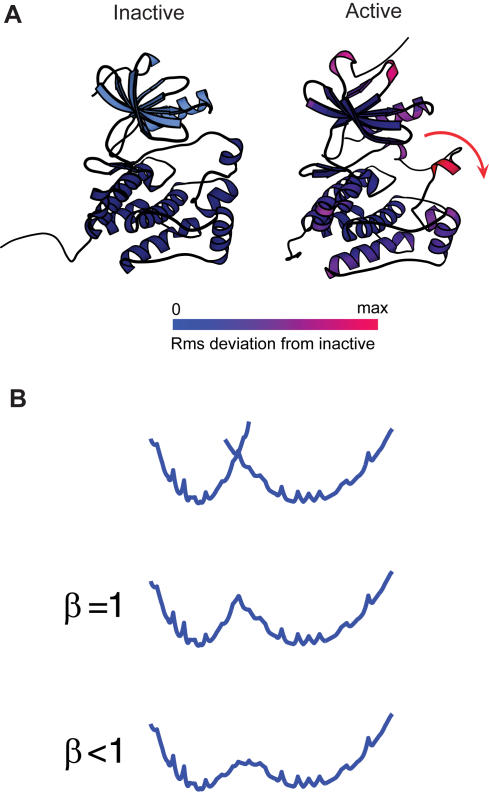
Experimental structures of the Src catalytic domain and cartoon representation for the multi-state model using switching by exponential averaging. (A) Crystallographic structures are taken from the inactive Hck (left, PDB ID: 1QCF) and the partially active c-Src (right, PDB ID: 1Y57), respectively [12],[15]. The primary conformational changes occur in a central activation loop (with Tyr416), as well as the relative orientation between the upper and lower portion (N-lobe and C-lobe), and the αC helix in the back. The color code in the active state (right) shows that the RMS-deviation from the inactive state for each residue. (B) A multi-state model: Switching by exponential averaging. Two reference structures supplied by the inactive and active Src are described by their own energy functions 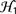 and 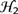 (see Materials and Methods). Then these two potentials are combined in a way such that they preserve the shape of energy surface near the energy minima while transitions are allowed between two minima, using an exponential averaging [33],[35]. The resulting energy function 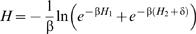 (Equations 1 and 5) encodes two experimental structures. The topological entropy of each reference structure is reflected by the width of the potential well. The adjustable parameter of β is used in simulations to tune the energetic barrier height to achieve a reasonable transition rate between two minima.

The nine members of the Src kinase family share a common structural organization, which consists of two regulatory SH3 and SH2 binding modules, followed by the catalytic domain [6]–[9]. A number of high-resolution crystal structures from three members of the Src-family (Hck, Lck, and c-Src) in different conformations have been captured, offering a great opportunity for a detailed view of the mechanism of allosteric regulation [10]–[15]. In its down-regulated inactive form, the three domains are assembled into an auto-inhibitory complex [10]–[12]. In its up-regulated active form, the complex is disassembled. The kinase catalytic domain is highly conserved among all protein kinases and its overall architecture resembles very closely that of other kinases such as protein kinase A [16]–[18] and Csk [19]–[21]. The catalytic domain comprises an N-terminal lobe (N-lobe) and a C-terminal lobe (C-lobe) (Figure 1). The active site is located between these two lobes, where the γ-phosphoryl group of ATP can be transferred to tyrosine residues of substrate peptides during the phosphorylation process [6],[22]. One important difference between the inactive and active form is the alternative conformations of the central activation-loop (A-loop), which controls accessibility to the active site [13],[15],[23]. In the down-regulated form, the A-loop is compact and blocks the active site to the substrate [11],[14]. Additional differences lie in the internal rotation of the αC helix and the relative orientation between the N- and C-lobes [24].

Structural studies of Src kinases by many groups have suggested some mechanisms for the regulation of the catalytic activity inferred from two “end-point” structures, although picturing how the protein dynamically switches from one state to another has remained elusive. One challenge for experiments to obtain the dynamic information is that the conformational switching process is inherently transient. Computer simulations based on physical models could provide a complementary approach to addressing these issues. To relate these static structures to the function, the dynamics of protein motion is required to fully monitor the conformational change process [25]–[28].

Theoretical studies based on standard all-atom simulations are prohibitive because the timescale of the transition is on the order of µsec [29]. A possible strategy to overcome timescale difficulties is to carry “targeted” or “steered” simulations [24], [30]–[32], though there is always the concern that the presence of nonphysical restraints may bias the transition pathway during the conformational change. This might be especially true when the transition involves multiple competing pathways. To overcome the timescale limitation of all-atom simulations and also avoid the nonphysical restraints used in biased simulations, we employ a coarse-grained model of Src kinase Hck. The model incorporates two individual experimental structures and allows switching between them. This is accomplished by using the recently developed multi-state model, or “two-state Gō model”, in which both experimental end-point structures are explicitly encoded in the energy function [33]–[41]. The present model differs from the “symmetrized-Gō model” used previously for studying domain swapping, in which the alternative conformation was implicitly in the monomeric conformation [42]–[44].

In the present study, we use this simplified model to explore the conformational activation of the Src catalytic domain. Notably, the regulatory modules SH2 and SH3 are not included in the present model. While the complete enzyme is obviously required to simulate the allosteric regulation mechanism, the activation process of the catalytic domain of Src, by itself, raises a number of important issues. For instance, the isolated catalytic domain is constitutively active [30], and it is plausible that it can adopt either the active or inactive conformation. For this reason, exploring the intrinsic dynamics of the isolated catalytic domain without its regulatory modules is of fundamental interest. The transition dynamics are simulated and characterized in the context of both a two-dimensional (2d) free energy landscape based on native contacts and a detailed structural network built from the simulations. The simulation trajectories are also mapped onto a discrete Markov model. Such a framework, proposed by Swope and collaborators [45], has been used to estimate long time-scale dynamics in protein folding. To test whether the Markov framework can be exploited in the context of an allosteric change, a similar analysis is performed for our coarse-grained simulations. Furthermore, the model suggests that there exist two parallel pathways, in one of which the conformational switching is coupled with local unfolding of the N-lobe of the catalytic domain. The results from this simplified model will serve as a first step toward understanding the thermodynamics and kinetics of conformational activation of the catalytic domain.

## Results/Discussion

### Model Description

To characterize the dynamical process of slow conformational changes involved in the Src catalytic domain activation, we construct a multi-state model with coarse-grained molecular representations [33]–[41]. Figure 1 shows the experimental structures of the kinase catalytic domain from Hck and c-Src, respectively, from which we first build and prepare the inactive and active states of the catalytic domain of Hck (see Materials and Methods). We create two energy potentials, corresponding to each of the reference structures, and combine these two potentials in such a way to preserve the shape of the energy surface near their own energy minimum while transitions are allowed between them. In practice, we adopt the strategy proposed by Hummer, Garcìa and collaborators [33],[35] and use an exponential averaging of two energy functions to construct the multi-state energy function 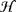 (Equations 1 and 5, and Figure 1; see details in Materials and Methods). The mixing parameter of β in Equation 1, which should not be confused with a physical temperature, is chosen to adjust the barrier height between two potential wells. All the parameters of the multi-state model are tuned to provide a quasi-realistic model of the Src conformational dynamics (see Materials and Methods). In summary, this simplified multi-state model takes into account the following factors: (i) the chain connectivity, (ii) the native contact interactions presented in two experimental structures, (iii) the excluded volume of each residue by using short-range repulsive interactions, (iv) the reference structures which, by definition, are the lowest-energy states, and (v) the conformational entropy reflected by the width of each potential well (Figure 1).

**Figure 2:**
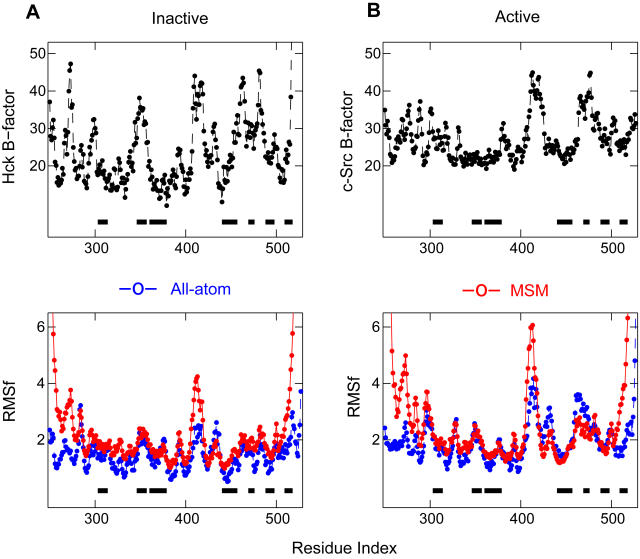
Semi-validation of the multi-state switching model. Comparison of thermal fluctuation between experiments, atomic simulations, and multi-state model (MSM) simulations. Shown are the data for the inactive (A) and active (B) states, respectively (top row). Experimental B-factors are taken from the full-length Hck and c-Src, respectively. For the active form, the Hck model structure was built from homology modeling of c-Src (see Materials and Methods). The RMS fluctuations (RMSf) (bottom row) were computed from the last 4 ns atomic simulations for the full-length Hck, and 10^9^-step MSM simulations with β = 1, respectively. Results show that the multi-state model correctly captures overall features of thermal fluctuation presented in both experiments and atomic simulations. For clarity, secondary structural elements of α-helices are indicated by black boxes.

As a semi-validation of the model, the RMS fluctuations for C_α_ atoms are computed from the coarse-grained simulations with the mixing parameter β = 1 and then compared with that from all-atom simulations with explicit solvent, and experimental B-factors of the corresponding crystal structures. Figure 2 shows that the multi-state model reproduces the experimental trend of thermal fluctuations for both inactive and active states, indicating that it is able to capture the basic features of the protein motion.

### Two-Dimensional Free Energy Landscapes

**Figure 3:**
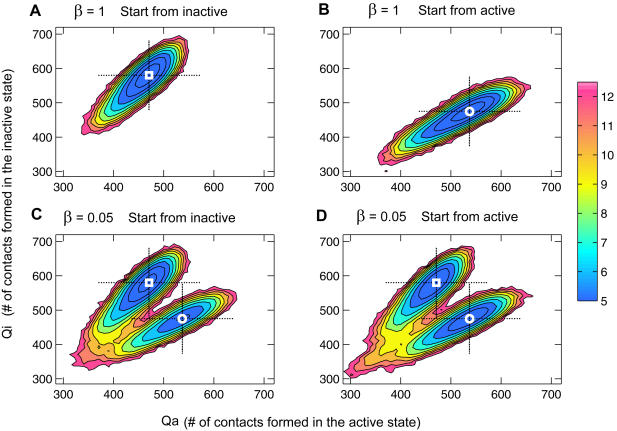
Free energy surfaces of Src conformational changes in the Src activation. Two-dimensional potentials of mean force *W*(*Q*_i_,*Q*_a_) are shown as functions of *Q*_i_ (the number of contacts made using the inactive state as a reference state) and *Q*_a_ (the number of contacts made using the active state as a reference). Each *W*(*Q*_i_,*Q*_a_) was computed from 100 µsec Langevin simulations with the multi-state model at 315 K. At a higher barrier (β = 1), the experimental structures are stable in their own minima (top row); at a lower barrier (β = 0.05), transitions occur between two minima (bottom row). The simulations were started with initial conformations in the inactive (left) and active (right), respectively.

To test the switching capability of this multi-state model, two-dimensional free energy landscapes are used to monitor the conformational changes. Two sets of simulations with mixing parameters of β = 1 and β = 0.05, respectively, are carried out to achieve different barrier heights between two energy minima. Figure 3 shows the 2d potentials of mean force (2d-PMF) *W*(*Q_i_*,*Q_a_*), where *Q_i_* and *Q_a_* are the number of native contacts formed using the inactive and active state, respectively, as a reference state. In this 2d projection, there are two free energy minima: one is the ensemble of the inactive state (Figure 3A) and the other is the ensemble of the active state (Figure 3B). With a high separating barrier (β = 1, Figure 3 top), the protein conformation stays within the local free energy minimum, since the barrier is too high to escape. As the barrier is lowered (β = 0.05, Figure 3 bottom), the free energy surfaces show that the catalytic domain can adopt alternative conformations corresponding to the two minima. To ensure that the system reaches the equilibrium, both the inactive and active conformations are used as initial conditions. Two free energy surfaces or *W* (*Q_i_*,*Q_a_*), each of which started with one of two starting points, are very similar, indicating that the simulations have converged and equilibrium is reached (Figure 3 bottom).

**Figure 4:**
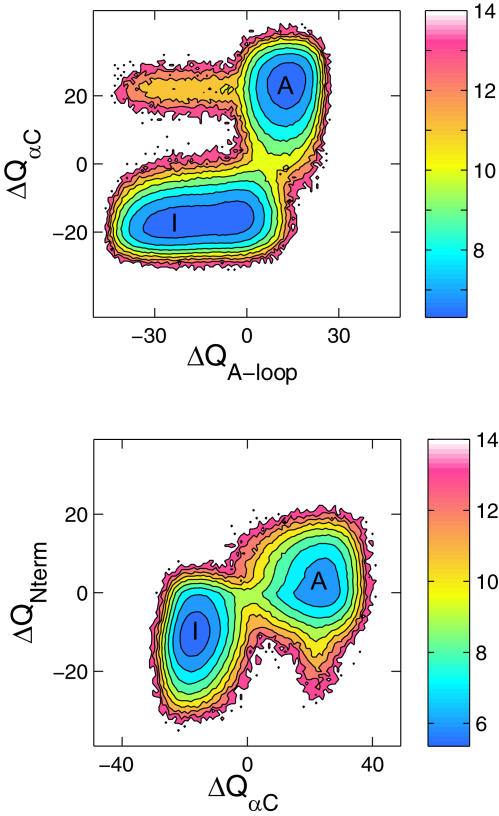
A switching mechanism observed from the simplified model for the αC helix, the A-loop, and the N-terminus. (Top) Two-dimensional PMF as functions of Δ*Q*_αC_ and Δ*Q*_A-loop_ indicates that the A-loop first opens up while the αC helix remain in the inactive conformation. This process is then followed by the αC helix rotation to adopt its active conformation. Residues I411 to P425 are used to define the flexible region of the A-loop. (Bottom) Two-dimensional PMF as functions of Δ*Q*_Nterm_ and Δ*Q*_αC_ suggests that the N-terminus is less restricted when the αC helix is in its active-like orientation. 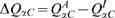 is defined as the contact difference between the inactive and active states. 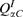 and 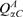 are the number of contacts made between any residue in the αC helix and any other residues for the inactive (I) and active (A) state, respectively. Similar definitions for Δ*Q*_A-loop_ and Δ*Q*_Nterm_ are used for the A-loop and the N-terminus, respectively. Residues P253 to L273 are used to define the N-terminus. The color bar in these contour plots represents the relative free energy in *k*_B_ *T*.

To further dissect the mechanism of the conformational transition we characterize the free energy landscape for the movements of important structural elements, namely the A-loop (part of the activation segment from residues A403 to T429 in c-Src numbering), the αC helix (residues V304 to K315) and the N-terminal region (residues P253 to L273). The order parameters, Δ*Q*_αC_, Δ*Q*_A-loop_, and Δ*Q*_Nterm_ are defined as the difference of the number of native contacts between the inactive and active conformation for the corresponding structural elements. This choice is appropriate for distinguishing different conformations for each structural element. The 2d-PMF W(Δ*Q*_αC_), shown in Figure 4 (top), indicates that the A-loop can fluctuate between an inactive-like conformation (Δ*Q*_A-loop_ = −30) and a near active-like conformation (Δ*Q*_A-loop_ = 0), while the αC helix remains very stable in the orientation of the inactive state. According to the free energy surface, the A-loop must first leave the inactive conformation before the αC helix is able to switch to its orientation in the active state. There is a larger barrier for the αC helix to rotate when the A-loop is in its closed inactive-like state. This two-step mechanism reported here is consistent with previously results obtained from umbrella sampling MD simulations with explicit solvent (Figure 3 in [24]). From a functional point of view, this suggests that the A-loop could easily fluctuate to conformations where it would be accessible for phosphorylation, while the αC helix is still in the inactive orientation. Previous work using umbrella sampling simulations also characterized the conformational freedom of the N-terminal end of the catalytic domain [31], suggesting that this region of the protein could be responsible for the bidirectional flow of allosteric information between the catalytic domain and the SH2 and SH3 binding modules. Specifically, it was shown that, when the αC helix was in the inactive orientation, the N-terminal was predominantly in an inactive-like conformation but could undergo fluctuations to the active-like conformation [31]. It was also shown that when the αC helix was in its active orientation, the N-terminal was then predominantly in an active-like conformation, but could also undergo fluctuations to inactive-like conformation. Here we check this notion with the simplified coarse-grained model. As shown in Figure 4 (bottom), the 2d-PMF as functions of Δ*Q*_Nterm_ and Δ*Q*_αC_ indicates that the N-terminal end is significantly less restricted than the αC helix, in qualitative accord with the previous results [31].

### The Structural Network: A Closer Look in a High-Dimensional Configurational Space

**Figure 5:**
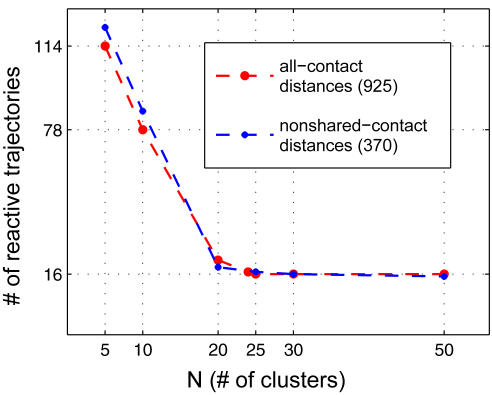
The choice of the number of clusters in the configurational space. The number of reactive trajectories is shown as a function of N (the number of clusters). Structural clustering was carried out based on pairwise C_α_ distances, by using the K-means algorithm implemented in MATLAB (see Materials and Methods). Two clustering schemes were carried out, using the C_α_ distances from all possible contacts present in both states (*Q*_1_+*Q*_2_+*Q*_shared_) and non-shared contacts (*Q*_1_+*Q*_2_), respectively. Both show that the number of reactive trajectories converges at the number of clusters of *N* = 25. A reactive trajectory is defined as one which leaves the inactive cluster (which the inactive conformation belongs to) and reaches the active cluster (which the active conformation belongs to).

In an attempt to provide a detailed picture of the topology of the conformational landscape, we use a graphic network analysis for Src conformational changes (e.g., [46]–[52]). The configurational space from all the simulation data with β = 0.05 (as shown in Figure 3 bottom) is discretized into a series of clusters. A total of 925 Cα pairwise distances, corresponding to all possible native interactions as defined in the energy function, is considered for partitioning the configurational space into *N* discrete clusters using a standard K-means clustering algorithm [53] (see Materials and Methods). The choice of the number of clusters was determined by examining the dependence of the number of “reactive” transitions (where the number of cluster is too small, the apparent number of transition is spuriously overestimated). A (forward) reactive trajectory is defined as one which left the inactive cluster and reached the active cluster. Figure 5 shows the number of reactive trajectories from the inactive to active state as a function of *N*. In the case of the Src catalytic domain, the configurational space can be divided into finer and finer clusters until the number of reactive trajectories is converged at around 16, when the cluster partition is *N* = 25. As a control, an additional set of K-means clustering analysis was performed with a fewer number of Cα distances restricted to those contacts that are not shared between the active and inactive states. Both confirm that the number of reactive trajectories converges at *N* = 25 as shown in Figure 5. For completeness, the clustering with distances from all possible native contacts was used for further analysis.

**Figure 6:**
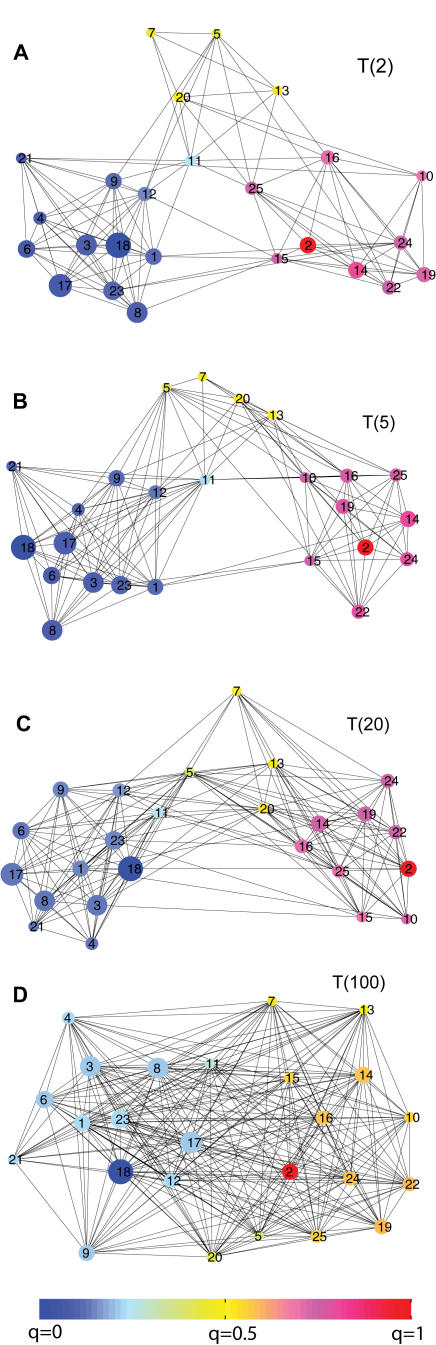
The structural network of Src catalytic domain conformational changes. Shown are the 2d force-directed layout of the networks of *T*(2), *T*(5), *T*(20), and *T*(100). The (forward) committor functions *q_i_* (Equation 6) (from inactive to active) for each cluster are shown by the color bar. Node 18 is the inactive cluster and node 2 is the active. *q*_18_ and *q*_2_ were set to be 0 and 1, respectively. The size of each node represents the cluster population as shown in Figure 13. For clarity, a cutoff of *L_ij_*>0.007 was used for the plot. The network of interconnecting clusters may be displayed as a 2d force-directed layout. Within this system, pairs of clusters (*i* and *j*, *i*≠*j*) are linked by elastic springs with spring constant 
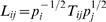
, where *p_i_* is the stationary distribution of any cluster *i* and {*p_i_*} is the eigenvector with unit eigenvalue of *T*. To achieve the 2d graphic layout, practically, we used a Monte Carlo search to find a local favorite combination, which resembles one state of the connectivity of these *N* interacting clusters (see Materials and Methods).

To visualize the detailed progress of conformational changes in the high-dimensional configurational space, a transition probability matrix is built among these *N* clusters as a function of a lag time *t* from the trajectories [54] (see Materials and Methods). From the transition matrix, one can construct a structural network to describe the conformational landscape (see Materials and Methods). Figure 6 shows the structural networks based on the transition matrix of *T*(*t*) at different lag times from *t* = 2 to 100 (in a unit of 5 ns). For the purpose of visualization, the size of each circle is linearly proportional to the cluster population in the simulations, and the distance between each pair of circles is inversely proportional to the interconversion rate between clusters. The circles are also color-coded according to the committor probability *q_i_* (from blue with *q_i_* = 0, to red with *q_i_* = 1), calculated within the context of a Markov model analysis (more details are provided below). There is a similar trend among these network layouts. Two ensembles of clusters, each of which has the reference state inside, are highly connected within their local minima, and some intermediate-state clusters lie in-between. When the lag time is small (e.g., *t* = 5), as required by the short-time properties for describing the local landscapes, *T*(*t*) gives rise to a robust connectivity of the network. When the lag time gets larger (*t* = 100), the clusters become highly connected because the kinetic information starts to be averaged out.

**Figure 7:**
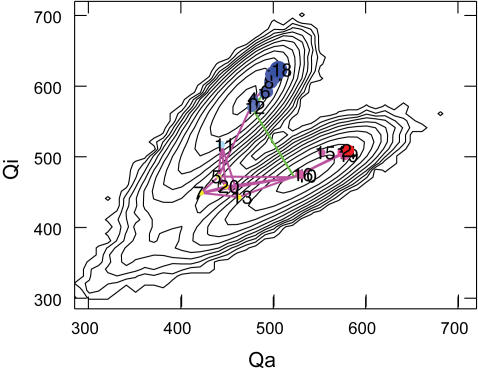
Projecting the structural network onto the 2d free energy surface. The 2d free energy surface *W*(*Q*_i_,*Q*_a_) was generated from a total of 200 µsec simulation data present in Figure 3 (bottom). The structural network was taken from the *T*(5) and the color code for each cluster is the same as in Figure 6. Each cluster falls nicely on top of the 2d free energy surface. Two representative reactive paths (as shown later in Figure 8) are also shown in green (Figure 8B) and magenta (Figure 8D), respectively.

It is possible to relate the high-resolution structural network with the 2d free energy surface. Figure 7 shows the projection of the network from *T*(5) into the W(*Q_i_*,*Q_a_*) (data from 200 µsec simulations with β = 0.05 as shown in both Figure 3C and 3D). As expected, each cluster in the network falls very nicely into its corresponding location in the 2d free energy surface, indicating that our construction of the structural network is consistent with the low-dimensional free energy surfaces or PMFs based on native contacts.

### Mechanisms of Src Conformational Activation

**Figure 8:**
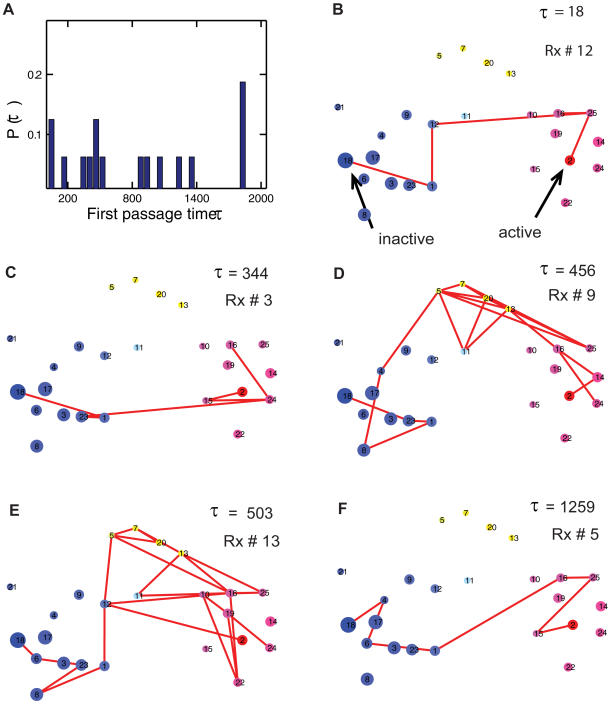
Reactive transition paths of the Src conformational activation. (A) The histogram of first passage times (τ) shows a broad distribution of a total of 16 reactive trajectories, implying there are multiple transition pathways. (B–F) Several representative reactive trajectories are projected on the network of *T*(5). All times shown here are in a unit of 5 ns. All the reactive paths readily suggest that there are two parallel transition pathways on the structural network.

To explore the transitions in configurational space, we examine all 16 reactive trajectories and projected them onto the structural network of *T*(5). Figure 8 shows the probability distribution of the first passage times from simulation trajectories ranging from τ = 18 to τ = 1859 (in a unit of 5 ns); the very broad distribution of the first passage times shows that there are multiplicity of pathways, each exploring different parts of the transition energy landscapes. We also project several representative reactive trajectories onto the network (Figure 8). It shows that actual realizations of reactive trajectories can be very diverse. Some go directly from the inactive to active cluster (Figure 8B, 8C, and 8F), and some take alternative routes by visiting the intermediate (yellow with *q_i_* = 0.5) clusters (Figure 8D and 8E). It also shows, clearly, even with direct transition without visiting the yellow region, the process could be very slow (τ = 1259, Figure 8F).

**Figure 9:**
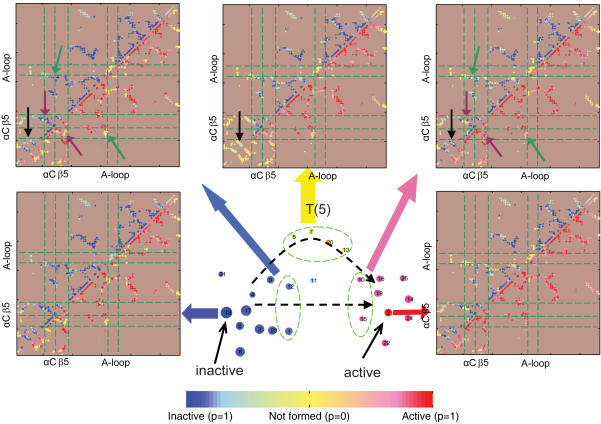
Mechanisms of the Src conformational activation. Shown are the contact probability maps for the selected ensemble of clusters from the network. Each dot in the map at (*i*,*j*) represents the interaction between residue *i* and *j* with contact formation probabilities *p* shown by the color bar: there are no contact interactions made if *p* = 0 (yellow); there are contact interactions made in either the inactive state or the active state if *p* = 1 (blue or red). The upper triangle is the probability map of contacts in the inactive state and the lower triangle is that in the active state. The highlighted regions are the A-loop (part of the activation segment from residues A403-T429), the αC helix (residues V304-K315), and the β5 strand (residues Y335-T338), representing three β strands in the N-lobe. Two parallel transition pathways can be identified from the reactive kinetic paths shown in Figure 8. In the first ensembles of paths, the inactive state directly switches to the active, including the A-loop opening (marked by green arrows) and the interaction switching among αC, A-loop, and β5 (marked by purple arrows). In the second ensembles of paths, there exists partial unfolding of N-terminal β-sheets (residues L267-M297) (marked by black arrows).

**Figure 10:**
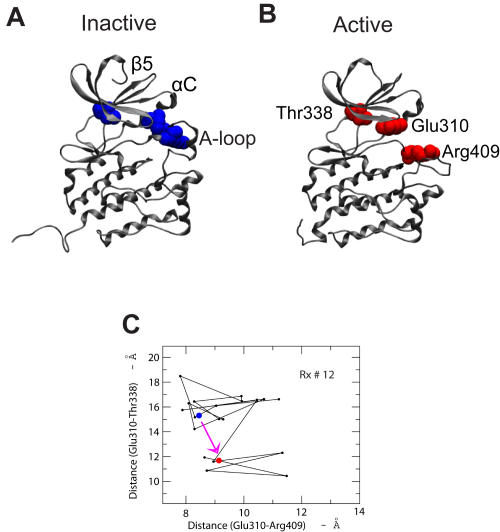
The switching of a network of representative interactions from simulations. Switching mechanism for the Src activation among the A-loop (residues 403–429), the αC helix (residues 304–314), and the β5 strand (residues 335–338) in the N-lobe. This can be represented by three highly conserved residues, Glu310, Thr338 and Arg409, where Glu310 exchanges interaction parters from Arg409 to Thr338 during the activation process from the inactive (A) to active (B) state. (C) One representative reactive path shows the interaction switch in the two-distance space (Glu310-Arg409, and Glu310-Thr338). The blue and red dots represent the inactive and active states, respectively.

Two parallel transition pathways can be assessed from the conformational landscapes and the reactive paths. The first pathway, represented by the ensemble of paths in Figure 8B, 8C, and 8F, displays direct transitions from the inactive to active state. The contact probability maps show that several locations undergo conformational changes upon activation (Figure 9). The first structural change taking place is an opening of the A-loop correlated with a loss of contacts with the αC helix (marked by green arrows in Figure 9). This can also be understood in the perspective of the 2d-PMF shown in Figure 4 (top). This initial process is followed by a loss of contact between helix αC and β strands in the N-lobe (e.g., β-strand 5 from residues Y335 to T338, marked by purple arrows in Figure 9). The latter movement may be viewed as mirroring the switched electrostatic network involving residues in β-strand 3 (residues T290 to M297) and αC, particularly between K295 and E310, which have been previously noted [32]. Here, these two processes are coupled (Figure 9). As suggested by Figure 10, the interaction networks between the helix αC (via E310), the A-loop (via R409), and the β-strand 5 (T338), β-strand 3 (K295) play an important role in the conformational transition upon activation [12],[14],[32]. This is consistent with experiments where a single residue mutation (T338 in c-Src and I338 in v-Src) destabilizes the inactive conformation [55]. Along this pathway, we also observe that a helix-coil transition occurs first in the solvent exposed region of the A-loop (residues N414 to A418), before all these interactions start to switch (Figure 9).

**Figure 11:**
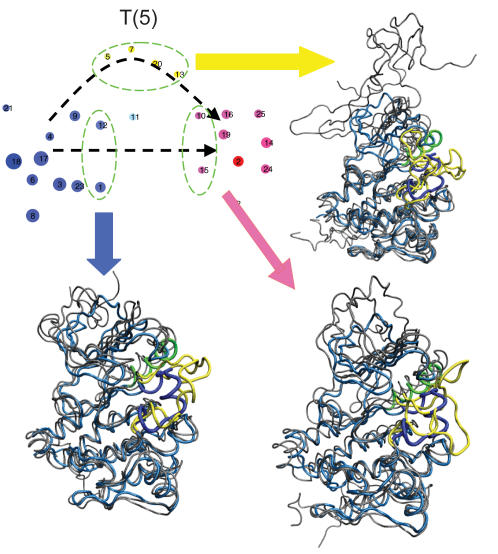
Structural features for Src catalytic domain activation. Ensembles of structures corresponding to selected clusters along transition pathways were used to illustrate the transitions. The inactive state is shown as a reference state in light blue. The activation loop is highlighted in yellow and the αC helix in green. The partially unfolding occurs in the β strands at the N-terminal region.

An alternative pathway is represented by an ensemble of paths, which crosses the intermediate-state clusters (e.g., Figure 8D and 8E). In this pathway, the lower portion (C-lobe) remains structurally intact, while a partial unfolding of β-sheets (residues L267-M297) in the N-lobe occurs as shown by the contact map (marked by black arrow in Figure 9). Figure 9 also indicates that this partial unfolding of the N-lobe region is coupled with the functional conformation changes in the A-loop, in contrast to the direct transition pathway where it remains folded while conformational transition takes place. This is consistent with the fact that both the conformational transition [29] and the β-sheet formation [56],[57] can take place on a timescale of µsec. In other words, the partial unfolding pathway, kinetically, could be competitive with more direct transitions (e.g., Figure 8B and 8F). There are indications that the two mechanisms might be coupled, as illustrated by the reactive trajectories in Figure 8E, in which the system travels back and forth and alternates its route between the first and second pathways. The structural features for these two pathways are shown in Figure 11, where the partial unfolding of the N-lobe is observed in the second pathway.

The notion that local unfolding may be linked to a conformation change playing a functional role is somewhat provocative, though it is consistent with previous results for adenylate kinase [34],[39] and for calmodulin [40]. Specific experiments could be designed to test this hypothesis in Src. For example, the relative propensity of the two pathways could perhaps be altered by changing the temperature, such that to alter which one dominates. In addition, it might be possible to use NMR hydrogen/deuterium exchange experiments [58]–[60] to detect the occurrence of partially unfolded intermediates during the conformational activation of Src.

### A Markov Analysis for Src Conformational Activation

Markov models can be used to harvest information from short time simulation trajectories and extrapolate to long timescale behavior [45], [61]–[65]. To test a Markov treatment in the case of the Src conformational changes, its ability to accurately describe the thermodynamics and kinetics was examined (see a brief summary for the Markov model in Materials and Methods). One underlying assumption of a discrete state Markov model is that the system should “forget” the state it came before making a transition to the next state. Failure to establish a lag time enabling one to satisfy this assumption may preclude the direct use of the model.

**Figure 12:**
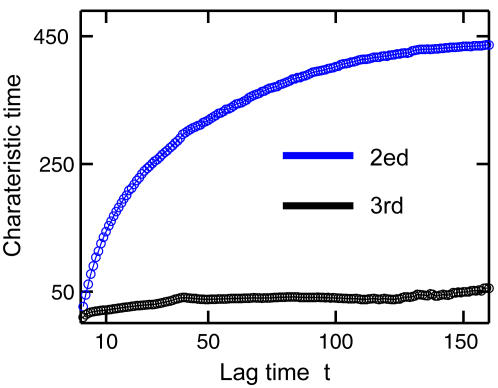
The characteristic time of the transition probability matrix *T*(*t*) Relaxation times (−*t*/ln λ) as functions of lag times *t* for *N* = 25. Shown are the second and third eigenvalues of the transition matrix *T*. The relaxation time approaches a constant around a lag time of *t* = 100 with a relaxation time of t^*^∼450. In the regime where *t*≪100, the system behaves as non-Markovian.

One necessary condition to test for this Markov behavior is to compute the second largest eigenvalue λ from the transition matrix *T*. If a process is Markovian, then the corresponding mode will exponentially decay as a function of the lag time *t* [45],[54]. Alternatively, the normalized relaxation time t^*^ = −*t*/ln λ should be nearly constant. As an indicator, the normalized relaxation time t^*^ provides a characteristic measure of the “memory” time needed to construct a valid Markov model. As shown in Figure 12, the time t^*^ approaches a constant of ≃450 around a lag time of *t*≃100. In the regime where *t*<100, the system behaves as non-Markovian. Often, the timescale for satisfying a Markovian behavior is beyond the accessible range of all-atom simulations, simply because t^*^ corresponds to the time of the motion associated with the slowest degree of freedom.

Assuming the system is Markovian, we analyze the transition probability matrix of *T*(*t*), especially with a short and atomic-simulation accessible lag time of *t*≪t^*^. Following Pande and collaborators (e.g., [61],[62]), we compute the forward committor *q^i^*, i.e., the probability of first reaching the active state before the inactive state having started from state *i* (see Equation 6, Materials and Methods). On the structural network, the *q_i_* effectively measure the “probabilistic distances” between cluster *i* and its destination. As already shown by the color bar in Figure 6, the computed *q_i_* (Equation 6) are projected onto the structural networks. Clearly, it helps to identify the cluster membership on the transition landscapes. For example, the clusters in yellow with *q_i_*∼0.5, indicating that they have a 50% chance of being reactive or non-reactive, sit in the middle of the allosteric transitions (Figure 6B). It is encouraging to note that, even though *q_i_* vary as the lag time changes, the ensemble of transition states consisting of the yellow clusters appears to be conserved across different networks, e.g., *T*(5) and *T*(20), indicating these relative distances yield a qualitatively robust measurement for identifying each cluster on the network.

**Figure 13:**
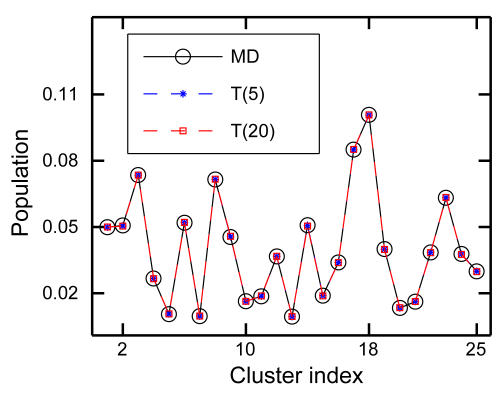
Thermodynamics from the Markov analysis. The equilibrium (or steady-state) population of all clusters (</emph>*N* = 25). Shown is the comparison between the cluster population from brute-force simulations and that from *T*(5) and *T*(20). The equilibrium distribution derived from the transition matrix *T* is equal to the true distribution from simulations. This is guaranteed by the construction of *T*(*t*).

Next, we compute the stationary population of each cluster *p_i_* by the eigenvector with unit eigenvalue of *T* as mentioned earlier (see Materials and Methods). Figure 13 shows the comparison of the cluster population between the computed *p_i_* and that obtained from the brute-force simulations. Within the non-Markovian regime (e.g., *t* = 5), the Markov model recovers the true thermodynamics in terms of the cluster population on the networks. This is expected, because the *T*(*t*) are built by enforcing detailed balance and time reversal, which guarantees that the stationary distribution should be directly taken from the population from the simulations.

**Figure 14:**
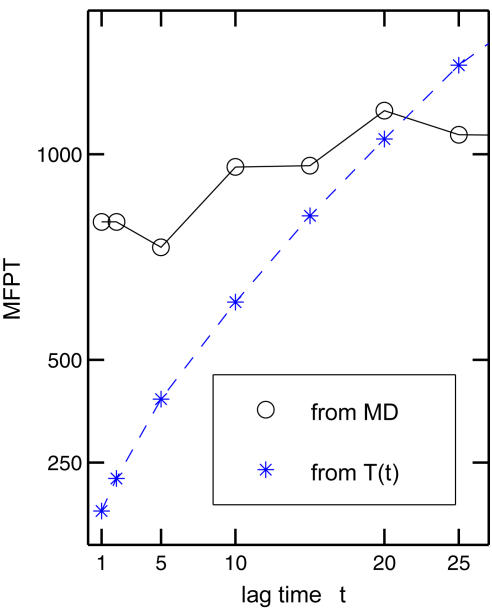
Kinetics from the Markov analysis. The mean first passage times (MFPT) as a function of lag time *t* at *N* = 25. The MFPTs calculated from the transition matrix *T* (Equation 7) do not fully reproduce the true transition time as obtained from the 16 reactive trajectories from simulations, e.g., at the short-time region *t*<10. The regime beyond *t*>25 is not shown because of the lack of sufficient number of reactive trajectories.

To test the kinetics prediction from the Markov model, we also compute mean first passage times (MFPT) τ*_i_* from *T* using Equation 7. Note that the definition of τ*_i_* used here is slightly different with what was used in [66]. Figure 14 shows the comparison between the MFPTs (τ*_A_*) obtained from brute-force MD simulations (marked by circles) and that computed from transition matrix *T* with the Markovian assumption (marked by stars). This result clearly indicates that the results from the Markovian analysis does not reproduce the transition time that obtained from simulations, as expected within the non-Markovian regime.

The current Markov model with macrostates based on a K-means clustering for the configurational space partitioning scheme is clearly limited. For instance, it does not successfully predict the long-time kinetics such as the mean first passage time. It is likely that the causes of this failure lie both in the lack of sampling from finite trajectories, and in the shortcomings of the partitioning scheme. Nevertheless, it is encouraging to note that the topology of the connected kinetic network of macrostates as well as the ranking with respect to committor probabilities are general features that appear to be qualitatively robust. For this reason, the Markov analysis, despite its limitations, remains a valuable tool to unmask several aspects of the transition pathways in the present system. We note that recent efforts have sought to develop strategies to enhance the Markovity and improve the performance of the rate prediction (e.g., [63],[64],[67]). Alternative approaches using a diffusive model have also emerged to address this issue (e.g., [57], [68]–[72]). It is possible that those developments will lead to more effective Markov analysis tools.

### Conclusion

The large-scale motions in the Src conformational activation take place on a relatively slow timescale that is beyond the reach of brute-force all-atom simulations. We develop a structure-based coarse-grained model to investigate the Src conformational changes on the free energy landscapes. We explore the detailed conformational landscape by clustering the configurational space and constructing a detailed structural network from simulations. We also test the performance of a Markov model in the cluster-partitioned space. Although the kinetics derived from the Markov model does not recover the true transition rate, the topology and connectivity of the kinetic network, inferred from the analysis seems to be robust. This important observation allows the identification of two transition pathways of the Src activation. A direct transition, a coordinated switch between a network of the A-loop, the αC helix, and β strands in the N-lobe, is coupled with an alternative pathway of partial unfolding of the N-lobe. The connectivity of the network appears to be qualitatively robust. The present results provide a broad framework for analyzing the conformational transition taking place upon Src activation. It is our hope that this framework shall guide the interpretation of experiments probing the dynamics of Src in solution, as well as additional simulation studies based on atomically detailed and coarse-grained models.

## Materials and Methods

### Preparation of the Hck Catalytic Domains

For the full-length Hck, the inactive state structure was taken from the assembled crystal structure (PDB ID: 1QCF); the active state structure was obtained from the partially active structure of c-Src (PDB ID: 1Y57) by sequence alignment (with 62% sequence identity) using ClustalW [73] and homology modeling using MODELLER [74]. These two structures were then solvated by a 150 mM KCl solution box and were simulated by NAMD 2.6 for 5 ns in the NPT ensemble [75]. Long range electronic forces were computed every two times steps by using the Particle Mesh Ewald algorithm with a time step of 2 fs. Atomic simulations were performed under standard conditions of 300 K with the all-atom PARAM27 CHARMM force field [76]. The structures of the isolated catalytic domain, in both inactive and active forms, were subsequently taken from the last frame of the all-atom simulations of the full-length Hck.

### The Multi-State Energy Function

Given two reference structures supplied by the inactive and active states of the catalytic domain, two independent structure-based potentials, 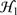 and 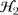, were first created at a simplified residue level. These two potentials were then combined in a way such that the shapes of the energy surface near their own energy minimum are preserved while transitions between two minima are allowed. We use a proposed approach based on an exponential averaging of 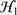 and 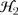, each of which describes one of the reference structures [33],[35],
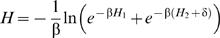
(1)The resulting energy function 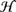 encodes two experimental structures of inactive and active states (see Figure 1). The parameter β is used to tune the energetic barrier height to achieve a reasonable transition rate between two minima. The parameter δ is the energy difference between two states. In the case of the Src catalytic domain, δ = −7 kcal/mol was used.

The energy functions for both reference states (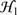 and 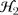) are defined as follows. We extended the structure-based (Gō-like) models [69], [77]–[81] to allow the switching occurs between two minima. The energy functions at the residue level, i.e., each residue is represented by its Cα atom, are
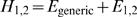
(2)where *E*_generic_ are the energy term presented in both 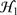 and 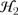, including the bond, angle, and dihedral interactions between adjacent residues, and the repulsive interactions for residue pairs that are not in contact in either active or inactive states,
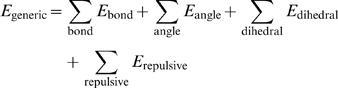
(3)The bond term *E*_bond_ = *k_r_*(*r*−*r_o_*)^2^, where *k_r_* = 100 kcal/mol and *r_o_* are the bond distances in the inactive state because the distance difference in two states is negligible (data not shown). The angle term 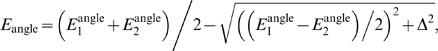, where 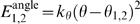. The angular force constant *k*_θ_ was set to 20 kcal/mol. θ_1_ and θ_2_ are the corresponding angles in reference structures. The coupling constant Δ is the coupling constant, Δ = *E_b_*−*E*_max_ and Δ = 0, if *E_b_*<*E*_max_, where *E_b_* = *k*_θ_((θ_1_−θ_2_)/2)^2^ and *E*_max_ was set to 0.5 kcal/mol. This angle term was chosen in this way such as the barrier height between two angles has a upper limit of *E*_max_, in a spirit that was used by Okazaki *et al.* [37]. The dihedral term 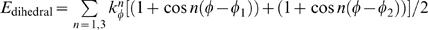, where 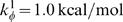 and 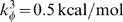. Note that the angle and dihedral terms in 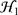 and 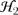 are generalized here to allow switching capability between two states. The repulsive term is for the residue pairs that are not in contacts in either inactive or active state, 
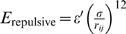
, where σ = 3.8 Å and 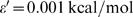. Residue pairs that make contacts in the reference states are modeled as a Lennard-Jones potential, *E*_1.2_, for the inactive and active states, respectively.
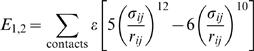
(4)where ε = 0.5 kcal/mol. σ*_ij_* are the distances between two contact-forming C_α_ atoms. Two residues (*i* and *j*) are determined to be form a contact if *r_ij_* was within a factor of 1.2 of the corresponding native distance after using the CSU package [82]. There are three types of residue contacts [37]: (1) those residue pairs that made contacts in both inactive and active states (shared contacts, *Q*_shared_ = 555); (2) those pairs that made contacts only the inactive state (nonshared contacts, *Q*_1_ = 192); (3) those pairs that made contacts only the active state (nonshared contacts, *Q*_2_ = 178). Therefore, the total numbers of contacts in the inactive and active states are *Q*_1_+*Q*_shared_ = 747 and *Q*_2_+*Q*_shared_ = 733, respectively. Further details on the standard Gō-like model can be found in previous publications (e.g., [42],[69],[77],[78],[83]).

Combining the two energy functions 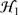 and 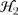 (Equations 1 and 2), the total energy *H* can be simplified into
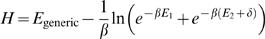
(5)Practically, this simplification, by exponential averaging over the contact energy terms only, will in principle allow us to implement this multi-state model into any molecular dynamics integrator.

### Simulation Implementation

The multi-state energy function was implemented in the molecular dynamics package CHARMM [84]. The Langevin dynamics simulations were carried out with a friction coefficient of 50 ps and a time step of 0.01 ps. The value of friction coefficient for Cα atoms was chosen to mimic the friction for the whole atomic-detailed residues. The simulations were carried out at a temperature of 315 K and snapshots were saved every 10,000 steps. If not specified, a time unit of 5 ns was used throughout for all multi-state model simulations.

### Structural Clustering by K-Means Algorithm

Following others (e.g., [85]), we used the K-means clustering algorithm [53] to partition the entire configurational space into *N* discrete clusters. This was done based on the Cα distances that made contacts in reference structure(s) for a total of 200 µsec simulations (data shown in Figure 3C and 3D). We used the two-phase iterative algorithm to minimize the sum of point-to-centroid distances as implemented in MATLAB. This procedure was repeated three times and the cluster partition with the lowest value of the sum of point-to-centroid distances was selected for the further analysis.

### Constructing Transition Probability Matrix *T*(t)

Among *N* clusters, we built a transition probability matrix *T*(*t*) = *T*(*j*,*t_o_*+*t*|*i*,*t_o_*), whose (*i*,*j*) element is transition probabilities of reaching cluster *j* after waiting for a lag time of *t*, given that the system was in cluster *i* at time *t_o_* [54]. In realistic molecular dynamics simulations, the detailed balance may not be strictly satisfied because of random sampling noise. Practically, we introduced the detailed balance and used time reversibility for the molecular dynamics simulations.

### Construction and Visualization of Structural Networks

To visualize the conformational changes in configurational space, a graph-like structural network may be constructed from the transition probability matrix *T*(*t*). The transition network among clusters may be viewed by a spring-and-charged *N*-particle system. Each cluster is assigned a unit charge and pairs of clusters (*i* and *j*, *I*≠*j*) are linked by elastic springs with spring constant 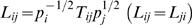, where *p_i_* is the stationary distribution of cluster *i* and {*p_i_*} is the eigenvector with unit eigenvalue of *T*. Therefore, the total energy of this spring-and-charged system includes Coulomb and spring-like interactions among *N* clusters. A Monte Carlo (MC) algorithm was used to find the local energy minimum and to obtain a 2d force-directed layout of the interacting network. A total number of 100 million MC steps were carried out for each layout.

### A Markov Analysis

Recently, a Markov model has been widely used to analyze MD simulation data (e.g., [45], [61]–[65]). If a process (or the transition represented by *T*(*t*) in this case) is Markovian [54], it has the following features. (i) As a main feature of the Markov model for time propagation, a Markov chain can provide the kinetic information from simulations, i.e., *T*(*nt*) = *T^n^*(*t*) where the process is still Markovian at a coarse-grainer time scale of *nt* [45]. (ii) As we mentioned earlier, the stationary distribution or cluster population of all *N* clusters is the eigenvector of the unit eigenvalue of *T*. (iii) The second largest eigenvalue λ provides the characteristic time τ (or the relaxation time constant of the single exponential decay) of the largest time-scale motion, t^*^ = −*t*/ln λ. (iv) The probabilities *q_i_* (the forward committor functions) of going from any cluster *i* to the final active cluster can be computed from *T* by
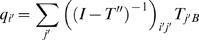
where *I* is an identity matrix and *T^″^* is the (*N*−2)×(*N*−2) matrix formed by removing the *A*th and *B*th rows and the *A*th and *B*th columns from *T*. 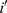 and 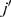 are the corresponding indices for *i* and *j* after removal. *A* and *B* are the inactive and active cluster indices, respectively, and *q_A_* and *q_B_* were set to 0 and 1, respectively. (v) Similarly, the mean first passage times τ*_i_* from any cluster *i* to the active cluster *B* can be computed by
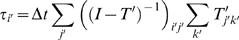
(7)where Δ*t* is the time unit when the transition matrix was built and is the (*N*−1)×(*N*−1) matrix formed by removing the *B*th row and the *B*th column. Here, 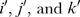 are the corresponding indices after removal. Therefore, the mean first passage time from the inactive to active cluster is τ = τ*_A_*.
